# Conscientious Objection: Understanding When and Why Primary Care Physicians Object to Providing Health Care to Transgender and Gender-Diverse Patients in an Appalachian Medical Center

**DOI:** 10.13023/jah.0601.05

**Published:** 2024-09-01

**Authors:** Mili S. Patel, Kelley A. Jones, Laura Davisson, Elizabeth Miller, Nicole Kahn, Pamela J. Murray, Kacie M. Kidd

**Affiliations:** University of Maryland, Baltimore; University of Pittsburgh; West Virginia University; Division of Adolescent Medicine, Department of Pediatrics, University of Pittsburgh, Children’s Hospital of Pittsburgh of UPMC

**Keywords:** Appalachia, barriers to care, gender-affirming care, health disparities, public policy and advocacy, transgender and gender-diverse patients

## Abstract

**Introduction:**

Transgender and gender-diverse (TGD) individuals face barriers to accessing primary and gender-affirming care, especially in rural regions where a national shortage of medical providers with skills in caring for TGD people is further magnified. This care may also be impacted by individual providers’ strongly held personal or faith beliefs and associated conscientious objection to care.

**Purpose:**

This study assesses the prevalence of conscientious objection to providing care and gender-affirming hormone (GAH) therapy to TGD individuals among physicians in an Appalachian academic medical center.

**Methods:**

An anonymous, online, cross-sectional survey of physicians was distributed to resident and faculty physicians in an Appalachian medical center. Survey domains included demographics, personal religious affiliations and practices, and assessments of willingness to provide specific types of care.

**Results:**

Surveyed physicians (n = 115) had no objection to caring for TGD patients but notable objection to prescribing GAH therapy to adults (23.5%) and minors (33.0%). Self-identified “very religious” physicians were more likely to object.

**Implications:**

Physician objection may present a barrier to care for TGD individuals in Appalachia. Provider and system-level interventions should be considered to ensure access to these necessary medical services.

## INTRODUCTION

Transgender and gender-diverse (TGD) individuals face significant barriers when attempting to access health care—both primary care services and gender-affirming interventions—due to a lack of healthcare providers (HCPs) who are adequately trained in providing care to this population, including the potential for denial of care due to gender identity.^1^,^2^ Social stigma and financial and socioeconomic concerns present additional barriers.^1^ Inability to access culturally competent care contributes to increased rates of negative health outcomes in this population when compared with non-gender-diverse individuals, including substance abuse, depression, anxiety, suicidality, certain types of cancer, diabetes, and cardiovascular diseases.^1^–^4^

Rural communities demonstrate worse health outcomes compared with urban counterparts due to cultural and socioeconomic factors and scarce medical resources.^5^ According to the 2020 U.S. Census, 20% of the population lives in rural America, while only 11% of physicians practice in these areas.^6^ Approximately one in six TGD individuals live in rural areas, making them more likely to face infrastructural barriers to obtaining health care, including poor transportation and social barriers such as lack of TGD community.^7^,^8^ A recent study demonstrated that TGD individuals who reside in the Appalachian Region have experienced these and other barriers. Participants reported average travel time to access care of 1.5 hours; a need to travel out of state to access care; fear of discrimination at their provider’s office; lack of insurance coverage for gender-affirming care; lack of access to desired gender-affirming care; and inadequate HCP knowledge of caring for TGD patients.^7^

The intersection of religiosity and medicine is an additional factor likely to affect care of people who identify as TGD compared with those who do not. “Conscientious objection,” a phrase initially associated with opposition to military conflict, is used to describe physicians who refuse to provide professionally accepted and legal medical care due to strongly held moral beliefs, often religious in nature.^9^ As “conscientious objection” is the language used most frequently in the literature to describe this phenomenon, the term is adopted here throughout.

Little is known about the prevalence of faith-based conscientious objection to the care sought and received by TGD individuals, many of whom are religious themselves,^10^ but a legal basis exists in the U.S. to deny care to TGD people based on religious objections.^11^ The Free Exercise Clause of the First Amendment prohibits the government from interfering with providers’ exercise of their religious beliefs, and this constitutional protection is augmented by the federal Religious Freedom Restoration Act of 1993 (RFRA), as well as numerous state-specific expansions.^11^–^13^ RFRA’s intention was to provide protections to people of faith, and this has been applied to conscientious objection and the provision of health care.^11^–^13^

Research is needed to understand: (1) the current prevalence of conscientious objection to provision of health care to TGD individuals among primary care physicians in Appalachia; (2) the interaction of religious beliefs with these objections; (3) differences in conscientious objection prevalence by training level, background, and age; and (4) how such objections may impact patient care, particularly in a medically underserved region. The aim of this cross-sectional descriptive study is to address these gaps in the literature by examining the phenomenon in an Appalachian academic medical center. Due to the sensitive nature of the study, the name of the center has been withheld here, as has the full name of its institutional review board.

## METHODS

A quantitative, cross-sectional, descriptive study of resident and faculty physicians from primary care fields was conducted in an academic medical center in Appalachia. Physicians were in the fields of internal medicine, pediatrics, combined internal medicine and pediatrics (Med-Peds), obstetrics and gynecology (Ob-Gyns), or family medicine. All participants gave their informed written consent, and data were collected over a two-month period in 2018. Participants were emailed information and a link to the 10-minute, web-based survey. No compensation was provided; however, resident participants were entered into a raffle for gift cards. This study was deemed exempt by the institutional review board.

### Survey Design and Deployment

The survey covered three domains: demographics; personal religious affiliations and practices, including self-identified religiosity; and objections to providing specific types of care. Religiosity was assessed on a four-point Likert scale from “very religious” to “not at all religious.” Objections to medical care comprised three domains: providing care to specific patient populations (e.g., “transgender patients”), prescribing pharmacologic treatments (e.g., “prescribing gender-affirming hormone therapy to adults”), and performing procedural interventions (e.g., “female sterilization”). For the purposes of this study, data specific to the provision of health care to TGD patients were analyzed. Participants were asked to indicate if they had no objection, objections due to religious reasons, objections due to non-religious reasons, or objections due to both religious and non-religious reasons. Individuals who indicated only religious objection were combined with those who reported both religious and non-religious objection as “any religious objection.” The survey items were both investigator-derived as well as adapted from a national survey of faculty physicians and a study of medical students.^10^,^14^ The survey was piloted by trainee and faculty volunteers prior to implementation. Participants could skip any question and return to previous questions prior to submission.

### Analysis

Descriptive statistics were used to characterize the sample. Chi-squared tests were used to identify differences in willingness to prescribe gender affirming medications by self-identified religiosity. If the chi-squared test was significant at the *p* < .05 level, post-hoc pairwise comparisons were conducted using Wald tests to identify between-groups differences. Post-hoc tests used the Bonferroni correction to account for the family-wise error rate when conducting multiple comparisons with the *p*-level set to .05. Stata software version 14.2 was used in all analyses.

## RESULTS

A total of 115 physicians completed the survey; approximately half of participants were resident physicians (50.4%) and half were female (48.7%; see Table 1). Nearly three-quarters of the sample (71.3%) were under age 40 years. In assessing religiosity, 22.6% identified as very religious, 33.9% identified as moderately religious, 21.7% identified as slightly religious, and 21.7% identified as not at all religious (Table 2).

Table 2 shows the results of analyses focused on objections to prescribing gender-affirming hormones (GAH). Since only one respondent indicated a non-religious objection to caring for TGD patients, this outcome was excluded from further analysis.

### Prescribing GAH to Adults

A total of 8.7% of respondents had religious objections and 14.8% had only non-religious objections to prescribing GAH to adults. The chi-squared test indicated significant differences by religiosity (χ^2^ = 18.78, *p* = .005). Post-hoc comparisons indicated that those who identified as “very religious” were significantly more likely to have any objection (i.e., significantly less likely to have no objection) to prescribing GAH to adults compared with those who identified as “not religious at all” (*F* = 13.50, *p* < .01) and “slightly religious” (*F* = 13.50, *p* < .01). These differences were driven by significantly greater proportion of “very religious” respondents who endorsed religious objections (F=7.50, p<0.01 for both comparisons).

### Prescribing GAH to Minors with Parental Consent

Overall, 8.7% of respondents had religious objections and 24.3% had only non-religious objections to prescribing GAH to minors with parental consent. The chisquared test indicates significant differences by religiosity (χ^2^ = 20.04, *p* = .003). Similar to prescribing for adults, post-hoc comparisons indicated that those who identified as “very religious” were significantly more likely to have any objection to prescribing GAH to minors compared with those who identified as “not religious at all” (*F* = 17.70, *p* < .01) and “slightly religious” (*F* = 10.69, *p* < .01), which was driven by the greater proportion of “very religious” respondents who had religious objections to providing this care (*F* = 7.50, *p* < .01 for both comparisons).

## DISCUSSION

Despite the notable levels of religious objection demonstrated in this study, the institution in which it took place is not religiously affiliated and is a safety-net hospital in a rural and medically underserved region. Limitations to gender-affirming care could have profound consequences among patients who experience limited access and resources as well as an excess of poor health outcomes. Only one participant objected to the provision of care to a TGD patient, which aligns with current legal opinions related to the illegality of discrimination based on gender identity,^2^,^10^ however, objection to providing routine gender-affirming care has the potential to cause harm. An additional level of distress that TGD individuals in the rural setting could experience is knowing that some HCPs cite their religious beliefs as reason to deny them care, when many members of the TGD population share those same religious beliefs. A recent study of lesbian, gay, bisexual, transgender, queer, and other sexual and gender minority (LGBTQ+) individuals in Appalachia, of whom 30% identified as TGD, revealed that faith is important to many members of the LGBTQ+ community.^11^ Study participants came from diverse faith backgrounds, and many describe losing relationships with their religious communities as a result of coming out, leading to psychological harm.^14^ Individuals who have faced rejection by their faith communities may subsequently experience rejection by their HCP for the same reason.

Being able to receive gender-affirming medical care, including GAH, is associated with reduced mental health inequities for TGD people.^15^ One study of 1,229 self-identified TGD individuals determined that transgender men living in rural areas, in particular, are even more likely to experience mental health concerns than their non-rural counterparts.^15^ While all of the study participants indicated their willingness to see TGD patients, nearly one-quarter of them indicated that they would not be willing to prescribe GAH to TGD adults, and nearly one-third objected to prescribing GAH to minors—even with parental consent.

Approximately half of those study participants who objected to providing gender-affirming hormone therapy to a minor, but not for an adult, were pediatric-trained, suggesting that additional barriers to access are present for youth. Much like adults, TGD youth who are not able to access gender-affirming care are significantly more likely to experience depression, anxiety, suicidal ideation, suicide attempts, and substance use disorder than the general pediatric population, but these health inequities are also reduced when young people can access GAH.^16^–^18^

This hesitancy to prescribe GAH to rural TGD minors is taking place in an environment where legislation targeting TGD youth is abundant. In 2022 and 2023, 22 states passed legislation limiting or banning the use of medications for TGD minors. These bans stood in opposition to current medical guidelines and available evidence of positive impact. In fact, the health system under study here resides in an impacted state.^19^ Further research is needed to understand how limitations or bans on care impact conscientious objection to providing other care to TGD youth that is still legally permissible.

Gender-affirming care is increasingly falling to primary care providers (PCPs), like those surveyed in this study, at a time when there is a significant shortage of these providers in rural America.^5^,^20^ Experts are encouraging PCPs to expand their skills through continuing education related to gender-affirming care, as many have had little to no formal training in this area.^3^,^21^–^23^ Studies examining the influence of provider training on objection to gender-affirming care are needed.

## IMPLICATIONS

To our knowledge, this is the first study to examine conscientious objection by HCPs to the provision of care to TGD individuals in a rural setting. This study is limited by being a single-center cross-sectional study and may not be reflective of even physicians within the health system who opted not to participate let alone other medical systems serving rural and medically underserved regions. Additionally, it was not possible to determine the nature of the non-religious objections, making it difficult to ascertain if these were also conscientious objections (i.e. due to an ethical reason). Questions about religious identity may be further limiting due to the desire to include a variety of options for faith practice based on stakeholder feedback during survey development. It is also possible that participants answered items in ways that are not reflective of their medical practices. Finally, it was beyond the scope of this study to disaggregate data based on residence status and/or training location of physicians. We recognize that different patterns may emerge had the study been able to explore potential differences in views between resident physicians and non-resident / international physicians. Future studies should consider potential differences in physician objection by faith practice and other characteristics, assess objecting physician practices related to caring for TGD patients, and more thoroughly explore the nature of non-religious objections.

The rates of objection to commonly needed medical interventions found in this study have the potential to disenfranchise many patients in rural areas without easy access to care, the means to transport themselves, or the monetary and other opportunity costs to see an alternate provider. Even “low” rates of objection may leave individuals without alternatives in resource-poor and rural communities. While some have argued that patients may need to be responsible for determining the services rendered by individual providers, this assumes providers and institutions are accessible and transparent to the lay public.^24^ Ideally, it is the responsibility of the medical system, particularly in rural or otherwise resource-limited areas, to create clear and patient-friendly information regarding access to legal and medically appropriate care so as to minimize harms associated with conscientious objection.

**SUMMARY BOX**

**What is already known about this topic?**

Transgender and gender-diverse patients experience barriers to receiving routine and gender-affirming medical care, including a shortage of knowledgeable providers. Conscientious objection on the part of medical providers in rural areas has the potential to further limit avenues for care.

**What is added by this report?**

Appalachian physicians in this study demonstrated levels of conscientious objection likely to worsen known health inequity for TGD people.

**What are the implications for future research?**

Further research is needed to identify ways to reduce barriers for TGD individuals seeking evidence-based care in rural areas given the multiplicative impact of a paucity of providers and this study’s identified rate of provider conscientious objection.

## Figures and Tables

**Table 1:** Demographic characteristics of study participants (n = 115) *

Characteristic	n (%)
**Gender**	
Male	58 (50.4)
Female	56 (48.7)
Other	0 (0.0)
Preferred not to say	1 (0.9)
**Race/Ethnicity**	
White	75 (65.2)
Asian/Pacific Islander	25 (21.7)
Hispanic	2 (1.7)
Black	2 (1.7)
Multiracial	1 (0.9)
Preferred not to say	10 (8.7)
**Age**	
20–29 years	38 (33.0)
30–39 years	44 (38.3)
40–49 years	13 (11.3)
50–59 years	10 (8.7)
60+ years	8 (7.0)
Preferred not to say	2 (1.7)
**Resident**	
Yes	58 (50.4)
No	57 (49.6)
**Residency Training**	
Internal Medicine	51 (44.4)
Pediatrics	24 (20.9)
Med-Peds†	15 (13.0)
Family Medicine	11 (9.6)
Ob-Gyn†	9 (7.8)
Other	5 (4.3)
**Religious Affiliation**	
Catholicism	31 (27.0)
Christianity	18 (15.7)
No Religious Affiliation	21 (18.3)
Islam	16 (13.9)
Protestantism	6 (5.2)
Hinduism	9 (7.6)

NOTES:

* Questions were not force response and allowed multiple selections for race and religious practice.

† Med-Peds = internal medicine and pediatrics; Ob-Gyn = Obstetrics and gynecology

**Table 2:** Objections to Prescribing GAH for Adults and Minors by Self-Identified Religiosity *

n (%)	Self-Identified Religiosity					

Care Provided	Total	Very Religious	Moderately Religious	Slightly Religious	Not at all Religious	Chi-squared statistic, *p*-value
	n = 115	n = 26	n = 39	n = 25	n = 25	
		22.6%	33.9%	21.7%	21.7%	
**Prescription of GAH for TGD adults**						χ^2^ = 18.78, *p* = .005

No objection	88 (76.5)	13 (50.0)	29 (74.4)	23 (92.0)	23 (92.0)	

Any religious objection	10 (8.7)	6 (23.1)	4 (10.3)	0 (0)	0 (0)	

Only non-religious objection	17 (14.8)	7 (26.9)	6 (15.4)	2 (8.0)	2 (8.0)	

**Prescription of GAH**† **for TGD**† **minors with parental consent**						χ^2^ = 20.04, *p* = .003

No objection	77 (67.0)	10 (38.5)	25 (64.1)	20 (80.0)	22 (88.0)	

Any religious objection	10 (8.7)	6 (23.1)	4 (10.3)	0 (0)	0 (0)	

Only non-religious objection	28 (24.3)	10 (38.5)	10 (25.6)	5 (20.0)	3 (12.0)	

NOTES:

* Results from questions regarding prescribing GAH for adults and minors by reason for objection and by self-identified religiosity.

† TGD = transgender and gender diverse; GAH = gender-affirming hormones
